# Editorial: Integration of multi-faceted techniques to improve stress tolerance to achieve food and nutritional security

**DOI:** 10.3389/fpls.2023.1327790

**Published:** 2023-10-31

**Authors:** Naeem Khan, Mohsin Tanveer

**Affiliations:** ^1^ Agronomy Department, University of Florida, Gainesville, FL, United States; ^2^ Xinjiang Institute of Ecology and Geography, Chinese Academy of Sciences, Urumqi, China; ^3^ Tasmanian Institute of Agriculture, University of Tasmania, Hobart, TAS, Australia

**Keywords:** crop productivity, stress tolerance, plant breeding, plant biotechnology, multi-omics

Global hunger has been on the rise since 2014, posing a significant challenge amidst a growing world population, set to reach 9.8 billion by 2050. To meet the demand for food, a 50% increase in crop production is essential. Currently, around 821 million people suffer from undernourishment, indicating a substantial risk in achieving the Sustainable Development Goal of eradicating hunger by 2030. Climate change exacerbates this issue, causing a 3.1%–7.4% decrease in global crop yields for every one-degree Celsius rise in temperature. Therefore, it is crucial to explore integrated approaches, including OMICS, GWAS, breeding, and agronomic techniques, to develop “Climate Resilient Cultivars.” While plant breeding and advanced agricultural technologies have bolstered food security, focusing on single traits or genes is insufficient. The integration of diverse strategies, such as phenotyping, OMICS, GWAS, marker-assisted breeding, CRISPR-based gene editing, bio-imaging, and agronomic interventions like foliar application and root treatment, is vital. By enhancing crop productivity, stress tolerance, and disease resistance, these methods offer promising avenues to address the challenges posed by increasing food demand and climate change. Having said that, this Research Topic was designed to collect recent developments in improving stress tolerance in plants.

A study by Nian et al. identified 20 B-box (BBX) genes, denoted as MaBBX genes, in *Melilotus albus*. These genes were categorized into five clades based on phylogenetic analysis, with similar conserved domains within each clade indicating potential functional similarities. Analysis of conserved motifs revealed common patterns within subfamilies. The distribution mapping showed nonrandom localization of BBX proteins across eight chromosomes, primarily amplified through segmental replication, signifying a key evolutionary process. Cis-element analysis predicted various stress-related elements in MaBBX promoters. Under salt, cold, and dark stresses, MaBBX genes exhibited differential expression patterns. Notably, MaBBX13 showed potential significance in abiotic stress tolerance. Protein structure predictions indicated nuclear and cytoplasmic localization for most MaBBX proteins, confirmed by transient expression assays. Detailed information, including gene names, IDs, protein lengths, molecular weights, and subcellular localizations, was provided for all 20 MaBBX proteins. These findings contribute valuable insights into the regulatory mechanisms of BBX genes in *M. albus*, especially under abiotic stress conditions, paving the way for further research in this area.

Ahmad et al. examined 12 root and biomass traits in a *Brassica napus* L. recombinant inbred line (RIL) population under low nitrogen (LN), low phosphorus (LP), and low potassium (LK) conditions. Significant differences in these traits were noted among the RILs, with high heritability and strong correlations between different treatments. Quantitative trait loci (QTL) mapping revealed 57, 27, and 36 loci associated with LN, LP, and LK conditions, respectively, which were integrated into 18 significant QTL clusters through meta-analysis. Four major QTL clusters involving 25 QTLs were identified, explaining over 10% of phenotypic variances, highlighting their role in cooperative nutrient uptake. Additionally, 264 genes within these clusters, showing high expressions in roots and genetic variations between parents, were identified as potential candidates. Phenotypic differences between the parents- Zhongshuang11 and 4D122, were significant, indicating diverse genetic effects on the traits. Positive and significant correlations were observed among various traits, suggesting genetic stability across different stress conditions. Using high-density SNP linkage mapping, 57 loci associated with 12 root and biomass traits were detected under LN conditions, with high phenotypic variance explained by these loci. Positive and negative additive effects were identified, emphasizing the parents’ importance in trait variation. These findings provide valuable insights into genetic mechanisms related to nutrient uptake and stress responses in *B. napus*.

The study of Yao et al. was focused on optimizing the photorespiration pathway in potato plants by introducing glycolate oxidase (GLO) and catalase (CAT) into chloroplasts. Proper regulation of this pathway is crucial for essential functions such as light protection and stress resistance. Transgenic lines expressing these enzymes displayed altered phenotypes, including shortened height, deformed leaves, and tubers, indicating imbalances in the glycolate shunt. DAB staining revealed higher H_2_O_2_ levels in transgenic leaves, impacting plant development. Despite some abnormalities, transgenic lines showed enhanced photosynthetic performance under certain conditions, with significantly increased net photosynthetic rates compared to wild-type plants. Photo-response curves demonstrated improved photosynthetic rates in transgenic lines under high light intensity, suggesting the potential for recovering CO_2_ from photorespiration. However, incomplete removal of H_2_O_2_ due to limited CAT activity in transgenic plants may contribute to abnormal phenotypes. The study highlights the importance of coordinated expression of GLO and CAT to establish functional photorespiration pathways and optimize plant responses to varying environmental conditions.

In another study (Li et al.), the non-specific lipid transfer proteins (nsLTPs) gene family in sugarcane (*Saccharum* spp.) was explored, focusing on the response to leaf scald caused by *Xanthomonas albilineans* (Xa) infection and exogenous salicylic acid (SA) treatment. The genome-wide analysis in *Saccharum spontaneum* revealed 71 SsnsLTP genes, with tandem and segmental duplications contributing to gene family expansion. Certain SsnsLTP proteins interacted with other proteins, indicating functional connections. Expression analysis highlighted genes like ShnsLTPI.8/10/Gb.1 as positive regulators in response to Xa infection in the resistant cultivar (LCP85-384) but as negative regulators in the susceptible cultivar (ROC20). Under SA stress, ShnsLTPIV.3/VIII.1 genes were upregulated in both cultivars, suggesting their positive role, while ShnsLTPGb.1 showed potential negative effects. Notably, ShnsLTPGb.1 had contrasting roles in Xa infection and SA stress responses. The study provides valuable insights into SsnsLTPs’ functions under different stress conditions, offering potential sources for developing stress-tolerant sugarcane varieties. Gene duplications and interactions with key proteins were observed, enhancing our understanding of sugarcane’s stress responses

Plant tissue culture, a technique used to identify and isolate bioactive compounds, holds significant industrial applications in the food, pharmaceutical, and cosmetics sectors. Various crops are propagated *in vitro*, offering sustainable solutions to agricultural challenges, and ensuring a year-round food supply. Plants are rich sources of medicinal phytochemicals, making them valuable for pharmaceuticals and nutraceuticals, as well as cosmetics, due to their anti-aging, anti-wrinkle, and other beneficial properties. *In-vitro* propagation offers advantages such as continuous food availability, conservation of endangered species, and efficient biosynthetic studies. Despite its advantages, challenges like continuous operation, product removal, and aseptic conditions must be addressed for large-scale applications. Techniques like CRISPR/Cas9 enable targeted genome engineering, creating new plant varieties without introducing foreign genes. Plant cell culture extracts are becoming popular in the cosmetics sector, offering effective, safe, and natural ingredients. Plant tissue culture methods show promise in developing novel active ingredients for cosmetics and skincare. In the future, these techniques may serve as sources of unexplored biological compounds and alternative bio factories for recombinant proteins. Continued advancements, especially in gene editing and environmental manipulation, are expected to enhance the potential of tissue culture techniques. Addressing constraints will be crucial for their sustainable industrial applications on a large scale (Hasnain et al.).

## Author contributions

NK: Conceptualization, Data curation, Project administration, Supervision, Visualization, Writing – original draft, Writing – review & editing. MT: Investigation, Methodology, Project administration, Validation, Visualization, Writing – review & editing.

